# The effectiveness of a self-made modular elastic compression device for patients with a fracture of the tibia and fibula

**DOI:** 10.1186/s13018-020-01678-7

**Published:** 2020-04-16

**Authors:** Lingyuan Zeng, Yongrong Wang, Dongdong He, Yao He, Yuze Wang, Xiaochun Wei

**Affiliations:** 1 Department of Orthopaedic Surgery, The Second Hospital of Shanxi Medical University, 382 Wuyi Road, Xinghualing District, Taiyuan, 030001 Shanxi Province China; 2 Department of Fashion and Design College, Donghua University, Shanghai, 200000 China

**Keywords:** Modular, Combined elastic compression device, Compression therapy, Fracture of tibia and fibula, ERAS surgery

## Abstract

**Background:**

To evaluate the effectiveness of a self-made modular elastic compression device for patients with a fracture of the tibia and fibula.

**Methods:**

Fifty-nine healthy adult patients with a unilateral fracture of the tibia and fibula were randomly divided into an experimental group and a control group. The experimental group was given the self-made combined elastic compression device for the compression treatment of the affected limbs after the operation. The main endpoints included the convenience, safety, and effectiveness of the self-made modular elastic compression device for patients with a fracture of the tibia and fibula.

**Results:**

There were 29 cases in the experimental group and 30 cases in the control group. There were no significant differences between the two groups in the general data: age, gender, fracture site, and cause of injury. The preoperative swelling elimination time was 3.3 ± 1.2 days, and the postoperative swelling elimination time was 3.1 ± 1.4 days in the experimental group; the preoperative swelling elimination time was 6.3 ± 1.2 days, and the postoperative swelling elimination time was 7.3 ± 1.2 days in the control group. The preoperative and postoperative swelling degree in the experimental group was shorter than those in the control group. The difference in the postoperative detumescence time between the experimental group (3.1 ± 1.4 days) and the control group (7.3 ± 1.2 days) was significant, and the total hospital stay was 8.1 ± 1.5 days in the experimental group and 13 ± 2.5 days in the control group with a statistical significance of *P* < 0.05. The change of discharge hemoglobin volume (11.2 ± 6.5 g/L) of the experimental group was lower than that of the control group (3.5 ± 1.2 days), the total drainage volume was 260 ± 50 ml, and the change of admission and discharge hemoglobin volume was 30.3 ± 10.4 g/L. Specifically, although the difference in the average hospital stay between the two groups was statistically significant, the difference was only 1 day, and the clinical difference was not significant. However, in the change of the cumulative drainage volume and hemoglobin volume, the experimental group that was given compression therapy was significantly lower than the control group with a statistical significance (*P* < 0.05). The pressure injury (4 cases) in the experimental group was significantly lower than that in the control group (8 cases) (*P* < 0.05).

**Conclusion:**

A modular combined elastic compression device in patients with a tibial and fibular fracture can significantly accelerate a patient’s rehabilitation, shorten the hospital stay, reduce blood loss, relieve the patient’s pain, and relieve the patient’s social-economic burden during recovery.

## Background

A tibia-fibula fracture is a clinically common multiple fracture located in the lower leg with four closed fascial compartments. Post-fracture hemorrhagic hematoma and increased vascular permeability of inflammatory reactions cause severe limb swelling. Therefore, a fracture of the tibia and fibula requires effective detumescence treatment; otherwise, there may be serious complications, such as failure of the primary closure of the incision, incision dehiscence, incision infection, osteomyelitis, compartment syndrome, and even nonunion and disability. For a fracture of the tibia and fibula requiring surgical treatment, early surgical treatment is performed and postoperative recovery is good. The concept of rapid surgical recovery includes using a perioperative multimodal optimization protocol through preoperative education, intraoperative and postoperative pain relief, heat preservation, early post-operative functional exercise, reducing bleeding, and optimizing surgical protocol, while a series of measures can reduce complications, reduce pain, shorten the hospital stay, and reduce costs so that patients can resume their normal lives and return to work as soon as possible.

Kehlet, a Danish surgeon, proposed the Enhanced Recovery After Surgery (ERAS) surgical concept in 2001 [1] using a multimodal optimization protocol in the perioperative period [2] through pre-operative education, intraoperative and postoperative pain relief, thermal preservation, early post-operative ambulation, and reduced bleeding. A series of measures to optimize the surgical protocol were to reduce complications, reduce pain, shorten hospital stays, reduce costs [3], and allow the patients to return to life and work as quickly as possible [4]. A fracture of the tibia and fibula is the most common fracture [5] with a rate of 13.7%, with an increasing number reported in clinical observations recently [6]. Tibia and fibula fractures are mostly associated with high-energy violent injury, often unstable, and are a comminuted fracture complicated with soft tissue injury; surgical treatment is beneficial to ensure that the patient can participate in activities early and avoid the complications of conservative treatment [7]. Early internal fixation and functional exercise are beneficial for fracture healing and good functional recovery [8]. However, a fracture of the tibia and fibula requires adequate soft tissue swelling to resolve in order to minimize surgical complications due to soft tissue swelling (edema, bleeding, or congestion) which can be a long process [9] and is not consistent with the fast-recovery surgical concept.

A previous study demonstrated that elastic bandage compression prevented excessive swelling and facilitated early rehabilitation of the affected limb [10]. However, in patients with fractures, limb elevation can lead to limb instability while increasing limb injury and pain. For this reason, we compared the differences in quick recovery between lower limb pressure and simple traction elevation in patients with a fracture of the tibia and fibula, using a modular and convenient combined elastic compression device developed by us. The purpose of this study was to evaluate the convenience, safety, and effectiveness of the self-made combined elastic compression device in the ERAS of patients with a fracture of the tibia and fibula.

We conducted this study to explore the difference of the Enhanced Recovery After Surgery (ERAS) effect between the application of the self-made modular elastic compression device on lower limb pressure and simple traction to elevate the affected limb in patients with a fracture of the tibia and fibula.

## Materials and methods

### Study subjects

Three hundred forty-three patients with a fracture of the tibia and fibula in our hospital were chosen to participate in this study from August 1, 2018, to February 28, 2019. According to the inclusion and exclusion criteria, 59 adult patients with a unilateral fracture of the tibia and fibula were selected and randomly divided into the experimental group and the control group. The ethics committee of our hospital approved this study and all patients signed informed consent.

### Inclusion criteria and exclusion criteria

Inclusion criteria are as follows: (1) patients who had a fracture of the tibia and fibula; (2) patients aged 20–60 years old; (3) patients with stable blood pressure control. Exclusion criteria are as follows: (1) patients who had diabetes or coagulation abnormalities; (2) patients who had a unilateral closed fracture of the tibia and fibula; (3) patients who had trauma or a surgical history on the healthy side and intact skin of both the lower limbs before the injury; (4) patients with severe anemia, hypoproteinemia, severe cardiovascular and cerebrovascular diseases, diabetes, etc.; and (5) patients with vasospastic diseases, a history of thrombosis, or deep vein thrombosis after admission for compression therapy.

### Methods

Braun-frame calcaneal traction was given to both groups before the operation. The experimental group was treated with the self-made lower limb compression device after the compartment syndrome was excluded. The degree of swelling after detumescence was mild (< 10%). Based on the fracture site, 9 cases in the experimental group and 7 cases in the control group were left and drained after being fixed with an open reduction plate, and 21 cases in the experimental group and 23 cases in the control group received closed reduction intramedullary nailing without indwelling drainage. The drain pullout time is when the drainage is less than 50 ml [11]. After the operation, the two groups were treated with a brace to fix the elevation and detumescence of the affected limb. The experimental group was given another self-made lower limb compression device again after the operation to compress the affected limb until 6 weeks after the operation. The specific application method of the combined elastic bandage compression device was as follows: select the buffer liner of the appropriate size and several external liner components of the compression elastic bandage; then, place the liner according to the positioning of the liner. The tension is adjusted appropriately from the distal ankle of the lower leg to the suprapatellar 20 cm [12] using several compression elastic bandages overlapping 1/2 of the liner from the inferior to superior in sequence. The same experienced clinician operated on the patients in both groups. Both groups were given active functional exercises such as isometric contraction of the quadriceps femoris, toe flexion, and extension movement. During the treatment of the experimental group, it was necessary to closely observe the skin sensation and peripheral blood supply at the distal end of the limbs, relieve the elastic bandage when the limbs suffered severe pain or numbness, and decide whether to continue the application of the compression therapy after assessing the blood supply in the limbs [13].

### Observation indicators

The 15 cm lower edge of the patella in a straight position was selected as the measurement point of the calf circumference [14] and the changes in the calf diameter compared with the diameter of the healthy side was recorded in both groups to evaluate the degree of soft tissue swelling [15], the time of pre-operative swelling and postoperative swelling, and the time of postoperative drainage withdrawal. The cumulative drainage volume, the difference between the hemoglobin level measured in the morning on the second day after admission and the hemoglobin level measured in the morning on the day of discharge, and the risk of lower limb deep vein thrombosis (VTE) were scored using the Caprini score [16]. The study was designed and routinely tested four times, respectively, on the day of admission and the day before the operation. On the day of the operation and the day of discharge, the special cases included sudden swelling of the lower limbs or venous filling. The Caprini score was taken four times or in special cases compared with the formation amount of the lower extremity vascular color Doppler ultrasound deep vein thrombosis on the day of discharge or in special cases, and the risk of thrombosis was compared between the two groups to assess the effect of the compression therapy. The visual analog scale (VAS) [17] was used to assess the subjective pain sensation of the subjects. The pressure injury stage of the pressure ulcer of the 2016 National Pressure Ulcer Advisory Committee was used to observe the local skin pressure damage of the affected limb and lower leg.

### Statistical analysis

We used the software program SPSS 20.0 (IBM, Chicago, USA) to conduct the statistical analysis. Continuous variables were expressed as mean ± SD. Discontinuous variables were expressed as a percentage (%). For two comparisons, each value was compared by a *t* test when each datum conformed to a normal distribution, while the non-normally distributed continuous data were compared using non-parametric tests. The counting data were tested by a chi-square test. A value of *P* < 0.05 was considered statistically significant.

## Results

### The general characteristics

In this study, there were 29 cases in the experimental group (22 males and 7 females; aged 22–59 years old, mean age 32.3 years; 10 cases of middle-upper 1/3 fracture, 12 cases of middle-lower 1/3 fracture, 8 cases of middle-lower 1/3 fracture of the tibia and fibula; 20 cases of stable fracture, and 10 cases of unstable fracture. The internal fixation with an open reduction plate was performed in 9 cases and the intramedullary nail was closed in 21 cases. There were 30 cases in the control group, 21 males and 9 females; aged 18–61 years old, mean age 32.9 years; 12 cases of middle-upper 1/3 fracture, 14 cases of middle-lower 1/3 fracture of the tibia and fibula, 4 cases of middle-lower 1/3 fracture; 18 cases of stable fracture, and 12 cases of unstable fracture.

**Fig. 1:**
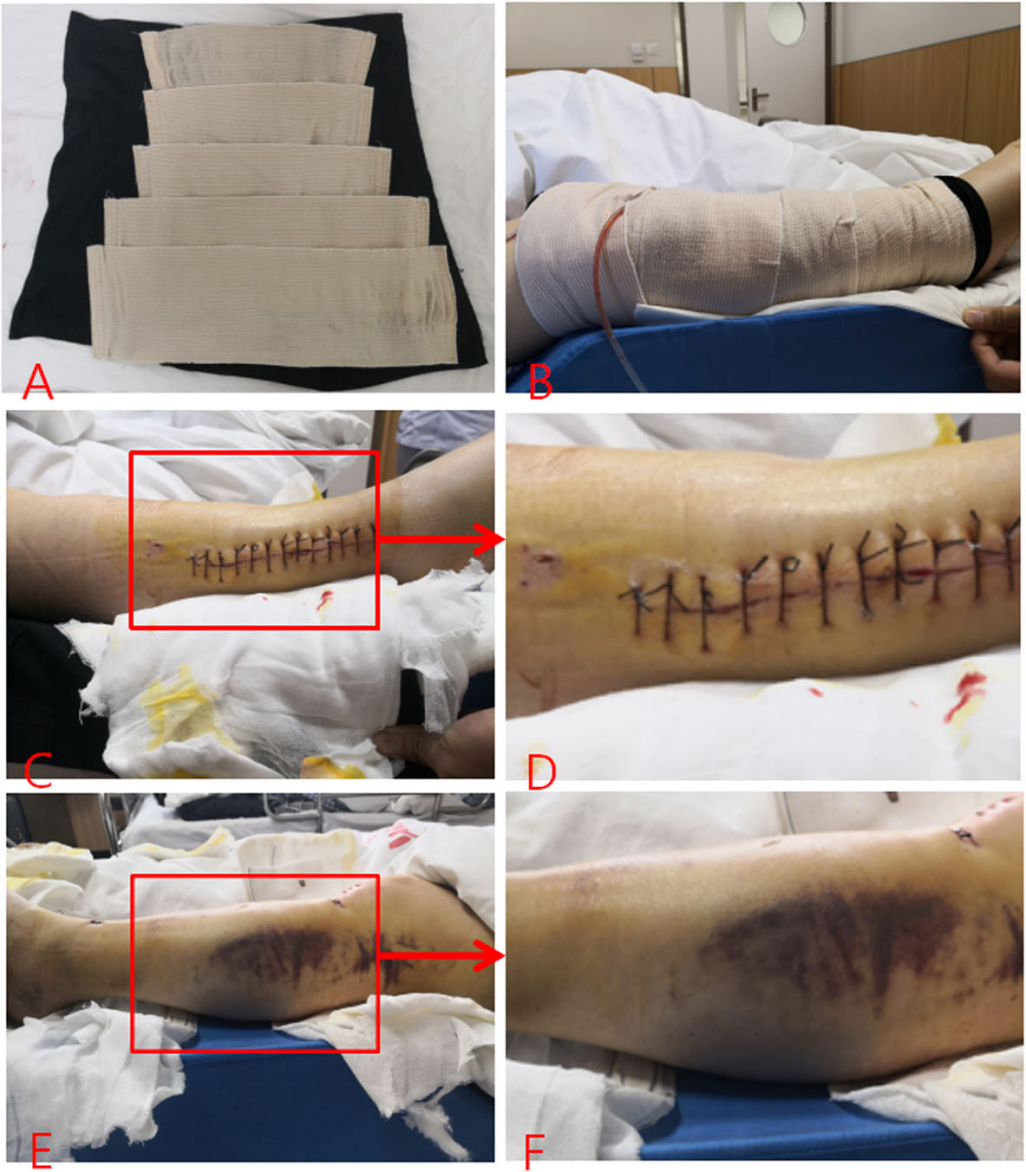
The comparison before and after treatment. **a** The modular combination of the lower extremity segmental compression device. **b** The experimental group after the placement of the lower extremity pressure device. **c** For the first day after opening the pressure device, the limb circumference diameter slightly increased and was relatively slender. **d** A part of **c**, no obvious subcutaneous ecchymosis, visible skin lines are still present. **e** The control group on the first day after opening the dressing, the peripheral diameter of the limbs increased significantly and was more swollen. **f** A part of **e**, obvious subcutaneous ecchymosis, the swelling is significant, and the skin is oily and bright.

There was no significant difference between the two groups in the general data: age, gender, fracture site, and cause of injury. There was also no infection in both groups. The preoperative swelling elimination time was 3.3 ± 1.2 days and the postoperative swelling elimination time was 3.1 ± 1.4 days in the experimental group; the preoperative swelling elimination time was 6.3 ± 1.2 days and the postoperative swelling elimination time was 7.3 ± 1.2 days in the control group. The preoperative and postoperative swelling degree in the experimental group was shorter than those in the control group. It is evident that the time to exclude compartment syndrome in the two groups was 3.2 ± 1.1 days, and the longest time to exclude compartment syndrome was 5 days, which was a case of high violence injury in a car accident of a comminuted fracture of the middle tibia and fibula, with the shortest time of one case being 1 day. A 55-year-old female patient (control group, 3.1 ± 0.9 days) suffered from a sprain while walking downstairs, and the longest was a patient with a mid-superior fracture of the tibia and fibula caused by high violence injury in a car accident for 6 days, and the shortest was also 1 day, which was a 30-year-old male patient with a transverse fracture caused by a fall during sliding on ice. There was no significant difference between the two groups (*P* > 0.05). One-half of the patients in group A (15 patients) entered the surgery on day 3 of the study, while only 4 patients in group B were available for surgery. Only 1 patient in the experimental group did not enter the surgery on day 5 of the study, while only 8 patients in the control group entered the surgery. The time from patient injury to surgery was 5.0 ± 1.1 days in the experimental group and 8.4 ± 2.1 days in the control group. It is also evident that under the precondition, there was no significant difference in the general data distribution between the two groups, the average hospital stay of the experimental group treated with moderate pressure was shorter than that of the control group by 3.4 days before the operation, and the preoperative detumescence time of the experimental group was more concentrated than that in the control group. The effect of compression therapy was clear in the experimental group. On the first postoperative day, the limb swelling degree of the experimental group was 11.1% lower than that in the control group (22.3%) and the swelling degree of the experimental group decreased. The control group decreased slowly after slightly increasing the swelling degree on the second postoperative day. The difference in the postoperative detumescence time between the experimental group (3.1 ± 1.4 days) and the control group (7.3 ± 1.2 days) was significant, and the total hospital stay was 8.1 ± 1.5 days in the experimental group and 13 ± 2.5 days in the control group with a statistical significance (*P* < 0.05) (Table 1) (Fig. 1).

**Table 1:** Comparison of the degree of soft tissue swelling between two groups (x ± s)

Time	Pre-operative					Post-operative				
	Test group		Control group			Test group		Control group		
	Participants	Degree of swelling (%)	Participants	Degree of swelling (%)	*P*	Participants	Degree of swelling (%)	Participants	Degree of swelling (%)	*P*
1 day	29	12.9	30	13.8	> 0.05	29	11	30	22.3%	< 0.05
2 days	23	9.7	28	14.4	< 0.05	29	7.0	30	23.7	< 0.05
3 days	14	8.3	26	15.7	< 0.05	17	6.5	30	20.5	< 0.05
4 days	4	5.6	25	10.5	< 0.05	9	4.9	25	17.6	< 0.05
5 days	1	5.1	22	7.3	< 0.05	6	3.5	18	16.4	< 0.05
6 days	0		17	6.8		1	3.4	12	15.2	< 0.05
7 days	0		2	6.4		0		5	11.6	
8 days	0		1	3.1				1	11.2	
Average admission days	3.3 ± 1.2		6.3 ± 1.2			3.1 ± 1.4		7.3 ± 1.2		

The beginning of the time is the first day after the exclusion of compartment syndrome. The degree of soft tissue swelling = (circumference of affected limb − circumference of healthy limb)/circumference of healthy limb × 100%, mild < 10%, 10 ~ 15% moderate, and severe > 15 (26)

### The blood loss during hospitalization

In terms of blood loss during hospitalization, the difference between the two groups was also evident. There was no significant difference in the number of patients with an indwelling drainage tube between the experimental group (*n* = 9) and group B (*n* = 7). The postoperative drainage pullout time (2.0 ± 0.8 days), cumulative drainage volume (120 ± 60 ml), and the patients in the experimental group were enrolled. The change in the discharge hemoglobin volume (11.2 ± 6.5 g/L) was lower than that in the control group (3.5 ± 1.2 days); the total drainage volume was 260 ± 50 ml, and the change of admission and discharge hemoglobin volume was 30.3 ± 10.4 g/L. In particular, although the difference in the average hospital stay between the two groups was statistically significant, the difference was only 1 day, and the clinical difference was not significant. However, in the change of the cumulative drainage volume and hemoglobin volume, the experimental group that was given compression therapy was significantly less than in the control group with statistical significance (*P* < 0.05) (Table 2).

**Table 2:** Comparison of blood loss between the two groups (x ± s, *n* = 30)

Group	Change in hemoglobin volume at admission and discharge (g/L)	Number of patients with indwelling drainage tube	Cumulative drain volume (ml)	Post-operative drain retention (days)
Test group	11.2 ± 6.5 g/L	9	120 ± 60	2.0 ± 0.8
Control group	30.3 ± 10.4	7	260 ± 50	3.5 ± 1.2
*P*	< 0.05	> 0.05	< 0.05	< 0.05

The change in hemoglobin volume at admission and discharge was all patients in both groups

### The pain of the patients

In terms of pain in the two groups, we found that on the first postoperative day, the visual analog score in the two groups was good (4.7 ± 1.1), which was lower than that in the control group (7.6 ± 1.4), and the difference between the two groups on the second postoperative day was poor (8.1 ± 2.2 in the experimental group and 9.3 + 0.5 in the control group); the difference was not significant. This may be due to nausea and vomiting caused by the pain pump on the second day after surgery. In general, we stopped using the pain pump on the second day and changed to oral pain medication. Subsequently, the pain score between the two groups showed a decreasing trend but the difference between the two groups gradually became noticeable. The patients in the experimental group reported significantly improved pain soon after 1–2 days of compression braking, while the patients in the control group still had significant pain for a longer period. The difference in the pain scores between the two groups was statistically significant (*P* < 0.05) (Table 3).

**Table 3:** Comparison of postoperative pain index between two groups (x ± s, *n* = 30)

Group	Day 1	Day 2	Day 3	Day 4	Day 5	Day 6	Day 7	Day 8
Test group	4.7 ± 1.1	8.1 ± 2.2	3.5 ± 1.2	2.2. ± 0.5	1.6 ± 0.3	1.5 ± 0.6	1.2 ± 0.5	1.0 ± 0.2
Control group	7.6 ± 1.4	9.3 + 0.5	6.9 ± 1.5	5.4 ± 1.6	4.6 ± 1.5	3.7 ± 1.2	3.2 ± 0.4	2.7 ± 0
P	< 0.05	> 0.05	< 0.05	< 0.05	< 0.05	< 0.05	< 0.05	< 0.05

Visual analog scale (VAS) (28) was used for the assessment of pain. Clinical evaluation divides “0 ~ 2” into “Excellent”, “3 to 5” into “Good”, “6 to 8” into “Fair”, “> 8” into “Poor”

### The pressure injury

The pressure injury in the experimental group (4 cases) was less than that in the control group (8 cases). Of these, 3 patients in the experimental group had stage 1 pressure injury as a high-energy injury caused by a car accident, and 1 patient with stage 2 pressure injuries had the longest time to exclude compartment syndrome, and the swelling degree of the affected limb was up to 31%. In the control group, there were 7 patients with stage 1 pressure injury and 1 patient with stage 2 pressure injury with a total of 8 cases. These 8 patients were in group B, including 3 patients with the highest degree of swelling, 3 patients with the highest age, 1 patient with both a higher age and degree of swelling, and 1 patient with stage 2 stress injury was a female patient with poor medical compliance during the pre-operative period of detumescence; the active quadriceps isometric contraction, toe flexion, and extensor motion were not actively performed and the patient did not actively change her position, resulting in stage 2 stress injury to the lateral malleolus of the affected limb. The patients with stage 1 pressure injury in the experimental group recovered after reducing the elastic bandage tension of the combined elastic compression device, and the device was not removed completely. The patients with stage 2 pressure injury in the experimental group healed after a dressing change and after removing the compression device. The patients with pressure injuries in the control group were mostly those with a high degree of swelling or older age. The time of postoperative detumescence was long before and after the operation, increasing the time of limb bed-rest compression. The time of injury was mostly 4–6 days before or after the operation, and the patients recovered after active treatment. The patients with stage 2 injuries in this group had external malleolus, the fracture site was located in the middle tibia, and the fibula did not require surgical treatment, which had no effect on the surgical field. Therefore, after significantly reducing the pain at the fracture site after surgery, it was feasible to cooperate with limb functional exercises to relieve compression, and then, the lateral malleolus healed 8 days after the surgery (Table 4).

**Table 4:** Comparison of pressure injury data between the two groups (x ± s, n = 30)

Group	Stage 1	Stage 2	Stage 3	Stage 4	Total
Experiment group	3	1	0	0	4
Control group	7	1	0	0	8
*P*	< 0.05				< 0.05

2016 National Pressure Ulcer Advisory Committee (NPUAP) on pressure injury divided into 4 stages (29): Stage 1: intact skin with erythema with constant digital pressure. Stage 2: partial cortical defect with dermal exposure. Phase 3: full-thickness defect of skin with exposed adipose tissue, usually visible granulation tissue or internal roll of wound edge, and local slough and/or eschar. Ulcers, with visible or palpable fascia, muscle, tendon, ligament, cartilage or bone exposed, may also have slough and/or eschar localized to the full thickness of the skin and tissue defects in Stage 4

### The risk of VTE

The risk of VTE in the experimental group was significantly lower than that in the control group. There was no significant difference between the two groups in the maximum score or special case score (*P* > 0.05). However, 3 cases of thrombosis in the experimental group were incomplete intermuscular venous thrombosis and all were found by routine vascular color ultrasound on the day of discharge. None of the cases had special conditions, while 8 patients in the control group had thrombosis, among which 4 patients had thrombosis detected by color ultrasound on the day before the operation and 1 patient was hospitalized for treatment of a fracture of the tibia and fibula after vascular surgery for popliteal vein thrombosis. In the control group, 4 patients had postoperative limb swelling, 3 patients had intermuscular venous thrombosis, and 1 patient had femoral venous thrombosis without obvious limb swelling, and there was a significant difference in thrombosis between the two groups (*P* < 0.05) (Table 5).

**Table 5:** Comparison of VTE risk assessment between the two groups

Group	Caprini highest score (x ± s)	Number of thromboses
Test Group	7.1 ± 1.6	3
Control Group	7.2 ± 1.5	8
*P*	> 0.05	

The 2009 version of the Caprini risk assessment model, which includes more than 40 different risk factors such as age, bed rest, surgery, history of thrombotic diseases, family history of thrombosis, etc., each risk factor is assigned 1–5 points according to different risk levels, respectively. According to the total score of risk factors of patients, the risk of VTE occurrence of patients is divided into 4 grades: low risk (0 ~ 1 point), moderate risk (2 points), high risk (3 ~ 4 points), and very high risk (≥ 5 points). Lower limb fracture is a very high risk factor (27)

## Discussion

Compression therapy, also known as pressure therapy, is the standard treatment of venous and lymphangitic diseases, achieving the prevention and treatment of skin scar hyperplasia and limb swelling by applying appropriate pressure on the body surface [18, 19]. Fabricine has been used in compression therapy for hand scar treatment in 1607 to promote hand function restoration, which is an earlier record of the use of compression therapy [20]. The clinical effects of pressure therapy mainly include the following [14]:(1) inhibition of scar hypertrophy, by changing the capillary of the hypertrophic scar and its blood flow state, preventing the growth of scar fibroblasts, effectively preventing and treating hypertrophic scars [21], and improving the appearance of skin scars, it can also prevent and treat joint contracture and deformity caused by hypertrophic scarring;(2) prevention and control of edema and alleviation of pain, it can promote blood and lymphatic reflux, reduce edema [22], and reduce pain due to decreased stimulation of the peripheral receptors by exudates; (3) promoting limb body contouring, it can promote the body contouring of an amputation stump and facilitate the assembly and application of prostheses; (4) prevention of deep vein thrombosis, pressure therapy can prevent the formation of deep vein thrombosis in the lower limbs of long-term bedridden patients [23]; and (5) the prevention and control of varicose veins of the lower extremity, it can prevent the occurrence of varicose veins of the lower limbs in people that are engaged in sitting or standing for a long time [24]. The use of an elastic bandage with appropriate pressure for tissue bandaging can reduce the production of lymphatic fluid in the tissue and leakage of fluid from the operation area, control exudation, errhysis, relieve swelling, and reduce postoperative pain of the patient. Meanwhile, it can also act as a pressure pump to accelerate the reflux of the lymph and deep veins, prevent thrombosis, etc. Therefore, it is recommended to apply an elastic bandage 10 cm from the plantar surface to the knee after relaxing the tourniquet to close the incision, which can effectively reduce the occult blood loss.

At present, elastic bandages, elastic sleeves, and elastic garments are commonly used for compression therapy [25]. To achieve uniform compression, other materials should be used in combination to overcome the irregular structure of the human body to achieve the curative effect. During the use of these bandages, the process of layer-by-layer winding installation and layer-by-layer disassembly of the elastic bandage is cumbersome, so it is difficult for a single person to independently operate; the application requires assistant or the patient to raise their limbs, increases the pain of the patient and increases the risk of fracture displacement and vascular, nerve, and soft tissue damage of the patient with the fracture. The self-adhesive elastic bandage could also not recover the prototype after use and the repeated use will lead to decreased elastic force. The clinical use of this technique mostly occurred once in previous applications, and the resources were wasted. The self-adhesive elastic bandage also slips off easily on the joint with a high range of motion or irregular shape of the limbs. The current compression therapy is mainly based on the experience of doctors to install the bandage on the patient under the most appropriate pressure, and there is no definite standard within the medical community. Therefore, its tightness cannot be accurately controlled [26], and the pressure cannot be adjusted according to the site after the layer-by-layer winding installation. If it is inappropriate to remove the re-dressing, it increases the economic burden, suffering, injury risk, and workload of the patients.

However, the existing lower limb elastic sleeve and elastic clothes are closed through a lateral zipper and the wear of the closed one shall be inserted from the distal end of the limb, which is not convenient for wearing. It is also necessary for the assistant to lift the limb off the table surface to avoid an increase in pain for the patient. For the fractured limb is unstable and cannot be used, the closure by a lateral zipper can avoid the shortcomings of an integrated closure, but it is also too large for the local surface to have a large amount of dressing to form the raised local tightening pressure after the operation so that the applied pressure cannot be adjusted according to the site.

The results of this study suggested that after the fracture of the tibia and fibula and compartment syndrome was excluded, the use of the combined lower limb compression device for postoperative rehabilitation could significantly shorten the preoperative edema and postoperative rehabilitation time of patients within the appropriate pressure range. The moderate pressure immobilization can also significantly reduce the pain and swelling sensation of patients and improve the comfort of treatment while moderate pressure immobilization also significantly reduces the preoperative and postoperative bleeding of patients, reduces the drainage indwelling time and, thus, reduces the risk of infection. More importantly, due to the reduced treatment time of the patient, the combined administration of moderate limb pressure can promote venous blood return and significantly reduce the risk of VTE in the patient. This combined lower limb compression device is made of high-elasticity material, which is comfortable and breathable with little influence on the flexion and extension activities of the lower limb joints and is designed as a modular placement and compression to achieve an individualized and flexible adjustment of the treatment pressure required by various parts.

However, there were several limitations to this study. Firstly, there is no clear clinical visual signs or symptoms to guide whether or when compression or detumescence treatment can be given for a fracture of the tibia and fibula limbs at the stage of swelling increase. Further experimental studies are needed to explore the signs or symptoms to guide the earlier administration of stress therapy. Secondly, there is no uniform pressure standard, and the risk of the bone compartment is higher than that in other sites due to the fracture of the tibia and fibula, each operator providing the application of specific bandage traction tension is given according to their own experience so it is difficult to develop a uniform standard. Thirdly, it is not possible to completely avoid excessive pressure damage and the ineffective treatment of too little pressure applied. Fourthly, it is necessary to further combine the characteristics of the high-risk bone compartment of a fracture of the tibia and fibula and further test the appropriate pressure range before and after the operation and the deformation range of the elastic bandage when pulling the pressure for a more convenient, safe, and effective application.

## Conclusion

A modular combined elastic compression device can significantly accelerate the patient’s rehabilitation, shorten the hospital stay, reduce blood loss, relieve the patient’s pain, and relieve the patient’s social-economic burden during recovery in patients with a tibial and fibular fracture.

## Data Availability

The datasets used and/or analyzed during the current study are available from the corresponding author upon reasonable request.

## Abbreviations

ERAS: Enhanced Recovery After Surgery
VTE: Vein thrombosis
VAS: Visual analog scale
